# Novel advanced patient-derived *in vitro* models of pediatric movement disorders to develop personalized therapeutic strategies

**DOI:** 10.3389/fneur.2026.1810863

**Published:** 2026-06-17

**Authors:** Scarlett Yeadon, Ainara Salazar-Villacorta, Manju A. Kurian, Serena Barral

**Affiliations:** 1 Department of Developmental Neurosciences, Zayed Centre for Research into Rare Disease in Children, GOS–Institute of Child Health, University College London, London, United Kingdom; 2 Department of Paediatrics, Donostia University Hospital, Biogipuzkoa Health Research Institute, Donostia/San Sebastián, Spain; 3 Department of Neurology, Great Ormond Street Hospital, London, United Kingdom

**Keywords:** *in vitro* model, induced pluriopotent stem cells (iPSC), organoids, pediatric movement disorders, therapies

## Abstract

Pediatric movement disorders (PMDs) are genetically and phenotypically heterogeneous neurodevelopmental and neurodegenerative conditions for which disease-modifying therapies remain limited, in part due to the poor translational relevance of traditional animal and cellular models. Recent advances in complex patient-derived *in vitro* systems, including regionally patterned neuronal cultures, organoids, assembloids, and microfluidic platforms, enable the study of human disease while preserving the patient's inherent genetic background. These models facilitate direct examination of pathogenic mechanisms, circuit-level dysfunction and therapeutic strategies, thereby offering new opportunities to advance precision medicine. This review synthesizes recent progress in patient-derived neural models relevant to PMDs, highlighting emerging technologies that increase biological and physiological complexity, and discusses key technical and translational challenges.

## Introduction

Pediatric Movement Disorders (PMDs) comprise a wide and heterogeneous group of neurological conditions in which disruptions of normal motor control lead to unusual movements, postures, or gait patterns. They are commonly divided into hyperkinetic disorders, characterized by excessive or unwanted movements or postures, and hypokinetic disorders, in which there are difficulties with the initiation of movement, and normal movement is reduced. Movement symptoms predominantly include, but are not limited to, dystonia, myoclonus, ataxia, Parkinsonism, tremor, and chorea (1). Many PMDs originate from rare single-gene mutations that occur during early neurodevelopment (Table 1).

**Table 1:** Representative genes associated with pediatric movement disorders, grouped by predominant clinical phenotype.

Predominant phenotype	Example genes *Gene name* (inheritance, molecular mechanism)	Comment
Dystonia	***ANO3*** [AD, GoF/DN (333)], ***HPCA*** [AR, LoF (334)], ***GCH1*** [AD/AR, LoF (335)], ***GNAL*** [AD, LoF/DN (336)], ***KMT2B*** [AD, LoF/DN (337)], ***THAP1*** [AD, LoF/DN (338)]	These genes often present as isolated dystonia; however, neurodevelopmental involvement (e.g., *KMT2B*) and additional movement disorders such as tremor (*ANO3*) or complex motor disorder (*GCH1*) are also common.
Chorea	***ADCY5*** [AD, GoF (339)], ***NKX2**-1* [AD, LoF (340)], ***PDE10A*** [AD, GoF (341, 342)]	Genes in which chorea is a core clinical feature. Chorea may also be observed as part of complex movement disorder phenotypes in other genetic conditions (e.g., *GNAO1, ATP1A3, PRRT2* and multiple metabolic disorders). Recessive forms of ADCY5 and PDE10A, likely related to LoF, have also been reported, usually associated with a predominant neurodevelopmental phenotype and chorea-dyskinesia.
Paroxysmal hyperkinetic movements	***ATP1A3*** [AD, LoF/DN (343)], ***KCNA1*** [AD, LoF (344)], ***PRRT2*** [AD/AR, LoF (345)], ***PNKD*** [AD, DN/LoF (346)], ***SLC2A1*** [AD, LoF (347)]	These genes are characterized by episodic hyperkinetic movements with a normal interictal neurological examination. Comparable paroxysmal phenotypes may also be frequently seen in *ADCY5* and *SCN8A*.
Tremor	***KCNN2*** [AD, LoF (348)], ***NOTCH2NLC*** [AD, GoF (349)], ***NUS1*** [AD, LoF (350)], ***TENM4*** [AD, DN (351)]	These genes are associated with early-onset postural or action tremor, usually associated with additional neurodevelopmental features (especially *NUS1, NOTCH2NLC, KCNN2*) rather than isolated tremor.
Myoclonus	***KCTD17*** [AD, LoF (352)], ***SGCE*** [AD, LoF (353)]	Both genes are most commonly associated with dystonia, with myoclonus representing a key clinical clue. In children, myoclonus should always prompt evaluation for epilepsy (e.g., *KCNC1, SCN1A*) and metabolic disorders (e.g., *POLG, CSTB, MERRF*).
Parkinsonism	***ATP13A2*** [AR, LoF (354)], ***DJ-1*** [AR, LoF (355)], ***PINK1*** [AR, LoF (355)], ***PRKN*** (AR, LoF) (355), ***WARS2*** [AR, LoF (356)]	Isolated Parkinsonism is rare in children and usually presents with bradykinesia and rigidity rather than rest tremor, frequently associated with dystonia. Childhood Parkinsonism represents a red flag for neurotransmitter disorders, particularly dopaminergic disorders (e.g., *DDC, SLC6A3, TH*, and BH4 pathway defects), and warrants targeted evaluation.
Ataxia	***ANO10*** [AR, LoF (357)], ***ATM*** [AR, LoF (358)], ***CACNA1A*** [AD, GoF/LoF (359)], ***FXN*** [AR, LoF (360)], ***ITPR1*** [AD, LoF/GoF (361)], ***SETX*** [AR, LoF (362, 363)], ***SPTBN2*** [AD/AR, DN/LoF (364)]	These genes are frequently associated with childhood-onset ataxia as the predominant phenotype. *CACNA1A* is associated with a broad phenotypic spectrum (including episodic ataxia and epilepsy). Some genes, particularly *SETX*, may present with overlapping phenotypes combining ataxia and motor neuron involvement, while others (*ANO10, ITPR1, SPTBN2*) are often associated with spasticity. *FXN* and *ATM* are multisystem disorders in which ataxia typically represents the predominant motor manifestation. When metabolic disorders present with an isolated or predominant movement phenotype, ataxia is the most common manifestation. Accordingly, in addition to *FXN, ANO10* or *SETX*, metabolic disorders predominantly presenting with ataxia, such as *COQ8A* or *POLG*, may also be included in this group.
Combined movement disorder	**Energy metabolism disorders:** ***COQ8A*** [AR, LoF (365)], ***ECHS1*** [AR, LoF (366)], ***PDHA1*** [XLD, LoF (367)], ***POLG*** [AR, LoF/DN (368)], ***SURF1*** [AR, LoF (369)] **Small molecule disorders:** ***ATP7B*** [AR, LoF (370)], ***DDC*** [AR, LoF (371)], ***PANK2*** [AR, LoF (372)], ***PTS*** [AR, LoF (373)], ***QDPR*** [AR, LoF (374)], ***TH*** [AR, LoF (375)] **Complex molecule disorders** ***ARSA*** [AR, LoF (376)], ***CLN6*** [AR, LoF (377)], ***FA2H*** [AR, LoF (378)], ***NPC1*** [AR, LoF (379)], ***NPC2*** [AR, LoF (380)], ***PLA2G6*** [AR, LoF (381)], ***WDR45*** [XLD, LoF (382)]	This group is predominantly represented by metabolic disorders and is characterized by complex and overlapping motor phenotypes, including dystonia, Parkinsonism, chorea, and ataxia, frequently associated with epilepsy or neurodevelopmental impairment. Non-metabolic genes, including *GNAO1, ATP1A3, ADCY5*, and *RHOBTB2*, may also give rise to similar complex motor disorder phenotypes.
Epilepsy	***CDKL5*** [AD, LoF (383)], ***FOXG1*** [AD, LoF (384)], **GNAO1** [AD, LoF/DN/GoF (385)), ***KCNC1*** [AD, DN (386)], ***KCNQ2*** [AD, DN/LoF (387)], ***KCNQ3*** [AD, LoF (388)], ***MECP2***, [*XLR/XLD, LoF* (389)), ***PCDH19*** [XL, LoF (390)], ***SCN1A*** [D/AR, LoF (391)], ***SCN2A*** [AD, LoF/GoF (392)], ***SCN8A*** [AD, GoF/LoF (393)], ***STXBP1*** [AD, LoF (394)]	These genes are primarily associated with developmental and epileptic encephalopathies, in which epilepsy represents the predominant clinical feature. Movement disorders, including dystonia, chorea, myoclonus, or paroxysmal hyperkinetic movements, are frequently observed as associated manifestations and may dominate the clinical picture at certain stages. In some cases, movement disorders may precede seizure onset or complicate epilepsy management.

AD, autosomal dominant; AR, autosomal recessive; DN, dominant negative; GoF, gain of function; LoF, loss of function; XL, X-linked; XLD, X-linked dominant; XLR, X-linked recessive. Bold text indicates disease-associated genes.

Traditionally, treatment strategies for PMDs are driven by clinical phenomenology rather than by the molecular causes of the disease. Symptomatic treatments can offer relief across a broad range of PMDs. For example, Levodopa (L-Dopa) has shown promise in improving dystonia in children with dopa-responsive dystonia and various other movement disorders (2–4). In selected cases, deep brain stimulation (DBS) has also proven effective, particularly in dystonias such as DYT-TOR1A dystonia or KMT2B-related dystonia (5, 6). However, reliance on symptomatic interventions presents substantial challenges for complex or multifaceted conditions. Patients may require multiple medications, each with potential side effects and interactions, and symptomatic therapies rare rarely curative. As a result, symptom management can become complicated, imprecise, and only partially effective.

PMDs are optimally positioned for precision therapies due to the combination of well-defined genetic causes of disease and early intervention opportunities. However, the development of personalized medicines for these disorders has been difficult, primarily due to a lack of suitable models that can capture allele heterogeneity and faithfully replicate phenotypes. While *in vivo* systems remain essential for assessing whole-organism physiology, systemic toxicity, and behavior, patient-derived models are increasingly recognized for their value in capturing human-specific gene regulation and neurodevelopmental timing.

This review examines how emerging patient-derived *in vitro* systems are transforming our ability to model PMDs, uncover disease mechanisms, and identify targeted disease-modifying therapies, and considers how these approaches may shape the future of treatment for other rare monogenic neurological disorders.

## Challenges in understanding and treating PMDS

The development of personalized therapies for PMDs has been limited due to several factors. First, the rarity and genetic heterogeneity of these conditions restrict patient recruitment and longitudinal or natural history studies, which have proven an essential component in the establishment of precision therapies for rare diseases (7). This delays the identification of robust genotype-phenotype correlations and clear clinical endpoints. Furthermore, while some disorders can be identified at birth via newborn screening, other PMDs manifest clinically years later or have overlapping phenotypes, making early detection and diagnosis challenging (8–10). Natural history studies therefore only include patients after genetic diagnosis, which for some early-onset PMDs can correspond to relatively advanced stages of disease (11). This consistent underrepresentation of the early disease trajectory phase is especially concerning, as this window is often when targeted treatment has the potential to be most effective (12, 13). This delay between early disease manifestation and clinical recognition is well-illustrated by cohort studies of childhood-onset PMDs. In a large tertiary-care series of 606 patients, mean symptom onset occurred in mid-childhood (approximately 7–9 years across phenotypes), yet diagnosis was typically made a decade or more later, with average delays of ~10 years for tic disorders and >11 years for other PMDs, including dystonia. Consequently, many patients are not formally diagnosed until adolescence or adulthood despite clear childhood onset (10).

In parallel, diagnosis is further complicated by clinical heterogeneity both among patients with the same disorder, and across broad range of PMDs. Certainty in diagnosis has often required genetic testing that, until recently, was not widely accessible (14). These challenges have led to delays in therapeutic intervention, promoting a reactive rather than a preventive clinical approach.

Furthermore, therapeutic development is inherently limited by the developmental nature of many PMDs, which often result from early, pre-natal disruptions in neuronal specification, migration, synaptogenesis, and circuit assembly (15). Once abnormal motor circuits form, targeted therapies may be less effective, particularly outside a neuroplasticity window (12, 16–18).

A further and often underappreciated challenge in understanding PMDs is the widespread phenomenon of reduced penetrance and variable expressivity. Reduced penetrance refers to the fact that not all individuals carrying a pathogenic variant develop clinical manifestations, whereas variable expressivity describes the broad range of disease severity, age at onset, and phenotypic features observed among affected individuals (19–22). This variability complicates diagnosis, prognosis, and trial stratification.

Both reduced penetrance and variable expressivity are influenced by the fact that many disease-relevant variants act as risk alleles rather than deterministic causes, with clinical expression shaped by genetic modifiers, epigenetic regulation, developmental timing and environmental factors (23–25). Well-established examples of reduced penetrance include maternal imprinting of the *SGCE* gene, which explains why individuals inheriting pathogenic variants from their mother typically remain unaffected in myoclonus–dystonia (26). Similarly, in DYT1 dystonia, the penetrance of the *TOR1A* ΔGAG mutation is markedly reduced, from approximately 30%−3%, by the presence of the D216H polymorphism acting in trans (27). Consequently, the introduction of a disease-associated variant in both *in vitro* and *in vivo* models may be insufficient to predict cellular phenotypes or recapitulate disease mechanisms in these systems.

Research progress has been further constrained by limited funding, reflecting the low prevalence of PMDs and the challenges of conducting adequately powered studies, although recent policy and funding initiatives are beginning to shift this landscape (28). Finally, access to human pediatric brain tissue is highly restricted, severely limiting opportunities to study disease-relevant human neural cells in their native developmental context. Consequently, early PMDs research has focused primarily on indirect approaches, including patient fibroblasts, genetically modified immortalized cell lines, and descriptive clinical observations or tests, rather than biological mechanistic and therapeutic studies in physiologically relevant systems.

## The need for advanced patient-derived systems

The emergence of patient-derived model systems has addressed many of the key limitations associated with animal and non-patient-derived/non-neuronal *in vitro* models of PMDs. While animal models do hold a particularly important role for translational research in tandem with *in vitro* models, their ability to accurately predict therapeutic efficacy could be constrained by species-specific differences in genetics, physiology, and gene regulation. Reliance on these models for translational decision-making could be actively harmful, as poor predictive performance may result in failed clinical trials, unanticipated toxicities, or the abandonment of therapies that might otherwise prove effective (29).

Species-specific differences have been well-documented for several PMD models. In DYT-TOR1A dystonia, mice carrying the human-identical heterozygous TOR1A ΔE mutation did not develop movement or behavioral abnormalities (30). Interestingly, however, confining *TOR1A* deletion to spinal cord and dorsal root ganglion neurons produced mice with remarkably similar phenotypes to those observed in patients, thus demonstrating region-specific disease phenotypic recapitulation (31).

Species-specific gene architecture can additionally prevent faithful modeling of human pathogenic variants. In aromatic L-amino acid decarboxylase (AADC) deficiency, the IVS6+4A>T variant activates a cryptic splice site, leading to a hypomorphic transcript. The mouse *Ddc* gene lacks this site, and the equivalent mutation instead causes exon skipping, creating a near-null allele. Consequently, knock-in mice exhibit only transient motor abnormalities and eventually become phenotypically indistinguishable from control mice, failing to model the persistent hypotonia, motor impairment, and developmental delay characteristic of human AADC deficiency (32, 33). Furthermore, a rat model of Kufor-Rakeb syndrome recapitulates some movement phenotypes, including a deficit in fine motor skills, though does not show spontaneous degeneration of the nigrostriatal pathway, a key pathological hallmark of disease manifestation in humans (34).

Simple *in vitro* systems present alternative issues. Primary human brain cells, while physiologically relevant, are limited by donor availability, high inter-individual variability, difficulties in extraction and culture, and short culture lifespans (35). Primary neural cells are also difficult to genetically modify due to their limited proliferative capacity and poor tolerance to genome editing, which limits their utility in mechanistic research (36).

The use of overexpression systems and the advent of Clustered regularly interspaced palindromic repeats (CRISPR)/Cas9-based genome editing have been transformative for disease modeling, enabling the precise introduction or correction of pathogenic variants, thereby greatly strengthening causal inference in mechanistic studies (37). However, the utility of these methods depends on the cellular system in which they are applied. Many genetically tractable models, including fibroblasts and immortalized cell lines such as HEK293 cells, are poorly suited to modeling PMDs. Fibroblasts lack the specialized molecular, electrophysiological, and developmental properties of neurons, while immortalized cell lines may exhibit aberrant gene expression, altered signaling pathways, and rely on artificial overexpression or knockout approaches that are not representative of native disease contexts (38).

Patient-derived induced pluripotent stem cell (iPSC) models and direct reprogramming technologies directly address many of the fundamental limitations that have historically constrained the study and treatment of PMDs. First, patient-derived *in vitro* models retain the complete genetic background of the donor, providing access to human neural material that encompasses both specific pathogenic variants and modifier alleles that may influence disease protection or susceptibility (39). In contrast to isogenic or overexpression-based systems, patient-derived models enable the direct investigation into the mechanisms underlying why a disease may manifest in some carriers, but not others. Furthermore, they facilitate the identification of protective mechanisms that could be exploited therapeutically.

Patient-derived models also preserve human-specific gene regulation, including alternative splicing, the use of alternative untranslated regions, and regulatory element activity (40). As gene-based therapies are a major therapeutic avenue for PMDs, models that accurately reflect human regulatory and transcriptomic contexts are essential. These platforms enable the development of personalized therapeutics, including allele-specific antisense oligonucleotides (ASOs), small interfering RNA, short-hairpin RNA, and gene-silencing or editing therapies.

A further advantage of *in vitro* systems is their capacity to model environmental perturbations which, given the marked clinical heterogeneity of many PMDs, may give rise to or exacerbate disease (41). Patient-derived *in vitro* models can be exposed to toxicants that may alter neurodevelopmental abnormalities observed across many PMDs, including heavy metals, maternal smoking-related toxins, plastic-derived chemicals, and persistent organic pollutants (42–50). A substantial collection of research has demonstrated that prenatal exposure to certain toxicants is associated with an increased risk of neuropsychiatric disorders, including autism-spectrum disorder, anxiety, depression, and schizophrenia (51–57). Interestingly, while PMD research has delineated aspects of how genetic/epigenetic factors may shape disease phenotypes, little work has been reported as to how exposure to environmental toxicant exposure may contribute to the complex and multifaceted presentation of PMDs with neuropsychiatric components, such as Tourette's syndrome (TS), Neurodegeneration with brain iron accumulation (NBIAs), Wilson Disease, DTDS, THD, ADCY5-related movement disorder, and cerebellar ataxias (58–64). Therefore, patient-derived *in vitro* models hold a significant advantage in their ability to model these potential environmental factors at a molecular level.

Advanced patient-derived *in vitro* models, including three-dimensional (3D) organoids, regionally patterned neural cultures/co-cultures, assembloids, and microfluidic/brain-on-chip platforms, can also recapitulate key aspects of human neurodevelopment, including early patterning, circuit formation, immune/glial interactions, and neuronal maturation. This enables the deeper interrogation of potential neurodevelopmental defects underlying the pathophysiology of PMDs, particularly those involving aberrant circuit formation or connectivity (65–68) (Table 2).

**Table 2:** Comparison of *in vitro* human neuronal models used to study pediatric movement disorders (PMDs) (69–71).

Model	Maturity	Cellular diversity	Reproducibility	Scalability/ throughput	Suitability for drug screening	Suitability for mechanistic studies	Key strengths	Key limitations
Directly reprogrammed neurons (iNs)	Low-moderate	Low	High	Moderate	Moderate	High	Rapid generation; partially retains donor age-related epigenetic signatures	Limited diversity; variable conversion efficacy
2D iPSC-derived neurons	Low-moderate	Low-moderate	High	Moderate-high	High	High	Patient-specific; scalable; amenable to genetic manipulation and screening	Developmental immaturity; limited circuit interactions
3D unguided brain organoids	Moderate-high	High	Moderate	Low-moderate	Low	Moderate-high	Recapitulate early human neurodevelopment; spontaneous tissue patterning	High batch variability; limited regional specificity and maturation
3D guided (patterned) brain organoids	Moderate-high	High	Low-moderate	Low	Low	Moderate	Enriched for defined neural identities; increased maturity;	Technical complexity; protocol variability, high batch variability
Assembloids	High	Very high	Low	Low	Very low	Moderate-high	Models inter-regional connectivity and network formation and migration; increased maturity	High experimental complexity; very low throughput; high batch variability
Microfluidic systems (organ-on-chip)	Moderate	Low-moderate	High	Moderate	Moderate	Moderate-high	Precise control of connectivity and microenvironment, vascular-like perfusion	Technically complex to generate; lack maturity
Vascularised organoids	High	High	Low	Low	Low	Moderate-high	Improved maturation, metabolic support, and tissue viability, BBB modeling	Technically demanding; limited standardization; protocol variability

During neurodevelopment, extrinsic and intrinsic mechanical forces play key roles in neuronal migration, differentiation, proliferation, and maturation (72). These forces arise from extrinsic structures and are converted into intracellular biochemical signals via mechanotransduction (73). During brain development, neurons and progenitor cells continuously sense and respond to mechanical cues such as tissue stiffness, Extracellular matrix (ECM) composition, and fluid dynamics, which regulate proliferation, migration, synaptogenesis, and the timing of neuronal maturation (74). Many PMDs arise from variants in genes involved in cytoskeletal regulation and membrane excitability, processes that directly intersect with mechanotransduction pathways (75–81). The perturbation of these mechanosensitive systems, through altered cytoskeletal dynamics, mechanosensitive ion channels, or ECM remodeling, can modify how disease-associated variants functionally manifest. These biomechanical cues in brain development are largely absent in traditional 2D cultures. 3D model systems and microfluidic/brain-on-chip platforms can incorporate this biomechanical context therefore include physical cues that may be relevant to the cellular and circuit-level manifestations of disease-associated variants.

## Advanced *in vitro* patient-derived models of PMDs

*In vitro* modeling of neurological diseases has evolved substantially over the past decade, with key advances in iPSC technology being particularly transformative for the personalized medicine field. iPSCs are generated by reprogramming somatic cells, such as fibroblasts or peripheral blood mononuclear cells, into a pluripotent state using defined transcription factors (82, 83). iPSCs can then be directed toward specific lineages through the application of developmental stage-appropriate signaling cues, enabling the generation of specific neuronal subtypes and glial populations (84).

Generating iPSCs has historically been achieved using several techniques. Viral-based approaches, such as Sendai virus, have a high success rate and have been employed extensively to generate large iPSC biobanks (85). However, cells must undergo multiple divisions to clear residual viral vectors, meaning the whole process can take many months and require significant human labor (86). These constraints have limited the high-throughput generation of patient-specific iPSC cohorts and, consequently, the widespread adoption of patient-derived disease models. More recently, key methodological developments and optimisations in episomal mRNA-based reprogramming techniques have improved both the efficiency and scalability of patient iPSC line generation. Patient-specific iPSC lines can now be generated within weeks and subsequently differentiated into disease-specific models, demonstrating the exciting feasibility of high-throughput therapeutic screening across a wide range of diseases (87).

A major strength of iPSC-based systems lies in the generation of isogenic control lines through CRISPR-Cas9-mediated genome editing, enabling pathogenic variants to be precisely engineered in either patients or control lines. Precise correction of pathogenic mutation in patient-derived iPSC allows for the control of specific disease phenotypes independently form genetic background. Insertion of pathogenic variants in a donor line, can allow for comparison of phenotypes across different mutations corrected or introduced in an otherwise identical genetic background (88). This approach enables the attribution of observed phenotypes to specific genetic mutations, effectively controlling for inter-individual genetic variability.

An alternative starting material for patient-derived models is fibroblasts, which can be directly converted into neural lineages without passing through a pluripotent state (89).

Thus, many different complex patient-derived *in vitro* models of PMDs have been developed, each suited to different aspects of disease research and translational applications. Based on their dimensionality, cellular complexity, and spatial organization, these models allow research from cell-autonomous neuronal phenotypes to multicellular interactions and circuit development, offering specialized platforms for the development of personalized therapies (Table 3).

**Table 3:** Complex *in vitro* neuronal models of PMDs as platforms for the identification of novel therapies.

Model platform	Disease	Experimental model	Observed phenotypes	Treatments tested	References
2D iPSC-derived regionally patterned cultures	AADC deficiency	iPSC-derived dopaminergic neurons	Reduced dopamine metabolism, impaired synaptic maturation, decreased neurite branching, reduced membrane capacitance	Protein replacement therapy, lentiviral delivery of wild-type *DDC*	(143)
	Tyrosine hydroxylase deficiency	iPSC-derived dopaminergic neurons	Decreased dopamine levels, impaired neurite arborisation, developmental stage-dependent maturation defects	L-DOPA supplementation at neuronal precursor or post-mitotic stage	(144)
	Spastic paraplegia type 5	iPSC-derived cortical projection neurons	Impaired axonal outgrowth, accumulation of axonal swellings, neurofilament disorganization, disrupted cholesterol metabolism leading to progressive axonal degeneration	Chenodeoxycholic acid supplementation	(149)
	GNAO1-related disorder	Patient-derived dorsal root ganglion and cortical neurons	Abnormal neuronal proliferation and differentiation, disrupted cell-fate commitment, dysregulated cAMP signaling, reduced basal intracellular Ca^2+^, impaired spontaneous neuronal activity	Allele-specific antisense oligonucleotide knockdown of mutant GNAO1 expression	(152)
	Dopamine transporter deficiency syndrome	iPSC-derived dopaminergic neurons	Impaired dopamine uptake, progressive apoptotic neurodegeneration	Lentiviral delivery of WT *SLC6A3*	(147)
	SCN8A-related epilepsy	iPSC-derived excitatory cortical neurons	Variant-specific sodium current abnormalities, axon initial segment shortening, prolonged action potential repolarisation, hyperexcitability	Sodium channel blockers (Riluzole)	(154)
Direct reprograming neurons	Myoclonic epilepsy and ragged red fibers (MERRF)	Directly reprogrammed induced neurons	Reduced neurite outgrowth, mitochondrial fragmentation, reduced membrane potential, elevated ROS, impaired autophagy	Rapamycin	(168)
	Rett syndrome	Directly reprogrammed induced neurons	Reduced dendritic arborisation, decreased VGLUT1 puncta, downregulated glutamate receptor genes, histone hyperacetylation	Protein replacement therapy (TAT-MeCP2)	(173)
	Friedreich Ataxia	Directly reprogrammed induced neurons	Reduced frataxin levels, iron accumulation, mitochondrial dysfunction	Alpha-lipoic acid supplementation	(180)
	DYT1–TOR1A dystonia	Directly reprogrammed motor neurons	Reduced neurite outgrowth, abnormal nuclear morphology, impaired nucleocytoplasmic transport, LMNB1 overexpression	shRNA-mediated LMNB1 knockdown	(184)
Neural organoids	CDKL5 deficiency disorder	Cortical organoids	Reduced progenitor proliferation, increased neuronal apoptosis, abnormal neuronal morphology, network hyperexcitability	Protein replacement therapy—Viral delivery of secretable TATk-CDKL5	(208)
	UBA5-associated encephalopathy (DEE44)	Cortical organoids	Reduced *UBA5* expression, impaired ufmylation, ER stress, loss of GABAergic interneurons, network hyperexcitability	SINEUP-mediated enhancement of *UBA5* translation and CRISPRa-dCas9 upregulation of *UBA5*	(68)
	Leigh syndrome	Cerebral organoids and 2D neural progenitor cells	Impaired COX assembly, defective mitochondrial respiration, disrupted neuronal maturation, abnormal cytoarchitecture, failure of metabolic shift to OXPHOS	AAV-mediated delivery of WT *SURF1*, bezafibrate-induced upregulation of PGC1A	(215)
	Tourette's syndrome	Cortical and basal ganglia organoids	Abnormal ventral patterning, reduced NKX2.1^+^ progenitors, impaired interneuron specification	None tested	(219)
	GNAO1-related disorders	Cortical organoids	Disrupted cortical organization, impaired neuronal differentiation, loss of polarized growth cone formation, Rho kinase hyperactivation	Supplementation with Rho-kinase inhibitor (Y27632)	(216)
Neural assembloids	SCN2A-related disorders	Cortico-striatal assembloids	Impaired cortical innervation of striatum, reduced dendritic spine density, AIS shortening, intrinsic neuronal hyperexcitability	CRISPRa-mediated upregulation of *SCN2A*	(226)
	Rett syndrome	Dorsal-ventral forebrain assembloids	Premature differentiation of neural progenitors, depletion of intermediate progenitors, reduced synaptic density, immature electrophysiology, impaired interneuron migration and integration	None tested	(232)
	22q13.3 deletion syndrome (Phelan–McDermid syndrome)	Cortico-striatal assembloids	Circuit-dependent hyperexcitability of striatal MSNs, reduced network synchronization, impaired cortico–striatal connectivity	None tested	(192)
Immune-competent organoids/assembloids	SCN2A-related disorders	Microglia-incorporated cortical, striatal, and midbrain organoids, and striatal-midbrain assembloids	Hyperexcitable striatal neurons, region-specific microglial transcriptional states, increased microglial calcium signaling in response to neuronal activity, hyperexcitable MSNs induced excessive microglial pruning of inhibitory synapses.	Pharmacological inhibition of microglial GABAB receptors	(245)
Microfuidics/organ-on-chip	Rett syndrome	3D microfluidic vascular model	Increased barrier permeability, reduced tight junction protein expression, altered secretion of vascular remodeling factors; endothelial miR-126-3p upregulation	Lentiviral delivery of antisense miR-126-3p	(302)
	Friedreich's ataxia	Compartmentalized DRG organoids in microfluidic axon-isolating devices	Reduced axonal mitochondrial density and size, increased mitochondrial fragmentation, impaired axon–muscle connectivity, defective muscle spindle-like structure formation	CRISPR/Cas9 excision of *FXN* exon 1 (removal of GAA expansion)	(303)

AIS, axon initial segment; MNS, medium spiny neurons.

## Neuronal subtype-specific vulnerability in PMDs

Across PMDs, pathogenic variants in specific genes confer selective vulnerability of discrete neuronal populations that are embedded within motor circuits. Voluntary movement is coordinated through distributed cortico-subcortical networks, principally the basal ganglia-thalamocortical and cerebello-thalamocortical circuits. Within the basal ganglia, excitatory cortical inputs to striatal medium spiny neurons (MSNs), together with dopaminergic modulation from substantia nigra pars compacta, regulate inhibitory output to the thalamus via the direct, indirect, and hyperdirect pathways, thereby shaping motor cortical activity (90). The type of complex model employed to interrogate disease mechanisms and assess precision therapeutics is supported by neuropathological findings for numerous PMDs.

Several neuronal populations have been identified as being specifically vulnerable in PMDs. In juvenile Parkinsonism, neuropathological analyses demonstrate a marked and preferential loss of dopaminergic neurons and gliosis in the substantia nigra pars compacta, particularly within the ventrolateral and medial regions, accompanied by α-synuclein pathology similarly to adult-onset Parkinson's disease (91). Similarly, a post-mortem study of tyrosine hydroxylase deficiency (THD) in with severe (“B-type”) presentation in a fetal brain identified pathologies related to dopaminergic neuron function, including reduced expression of TH, vesicular monoamine transporters, and dopamine receptors. In parallel, markers associated with GABAergic interneurons and glutamatergic projection neurons were also altered, alongside a widespread reduction of synaptic, axonal, and dendritic proteins despite preserved neuronal volume (92). In dopa-responsive dystonia linked to the DYT14 locus, dopaminergic neurons in the substantia nigra and noradrenergic neurons in the locus coeruleus were shown to be preserved in number, but exhibited profound hypomelanisation, with pigment loss showing asymmetry and preferential involvement of the ventrolateral substantia nigra, a region typically vulnerable in Parkinsonian neurodegeneration (93).

Both Friedreich's ataxia (FA) and ataxia telangiectasia (AT) can be grouped within hereditary cerebellar ataxias. Post-mortem studies in patients with FA demonstrate severe hypoplasia and progressive degeneration of large sensory neurons in the dorsal root ganglia, and large glutamatergic projection neurons in the cerebellar dentate nucleus with secondary transsynaptic loss of neurons in the posterior thoracic nucleus and degeneration of ascending sensory tracts (94, 95). Similarly in FA, studies demonstrate a loss of Purkinje cells and atrophy of the dentate nuclei in the cerebellum. In the spinal cord, atrophy of anterior horn motor neurons has been observed in addition to demyelination of the gracile fasciculi, responsible for carrying proprioceptive sense from the legs. Interestingly, findings also demonstrated cerebral vasculature abnormalities, with spongiform degeneration and white-matter injury occurring preferentially around structurally altered blood vessels. These data support the neuropathology of cerebellar ataxias also involves spinal motor pathways and ascending sensory systems, consistent with broader multisystem vulnerability (96).

Neuropathologic investigation of NBIA disorders demonstrates common patterns of degeneration, with prominent neuronal loss and iron accumulation in the globus pallidus and substantia nigra, associated gliosis, and frequent neuroaxonal spheroids. Although basal ganglia pathology predominates, cortical, cerebellar, brainstem, and spinal cord involvement is variably present across subtypes, often accompanied by tau and/or α-synuclein pathology (97, 98).

Within the basal ganglia, a joint study investigating ADCY5- and PDE10A-related movement disorders revealed convergent abnormalities within striatal MSNs. *In vivo* molecular imaging demonstrated reduced PDE10A expression in the striatum and globus pallidus, alongside reduced dopamine transporter availability and loss of neuromelanin-containing neurons in the substantia nigra, indicating involvement of both post-synaptic striatal neurons and presynaptic nigrostriatal dopaminergic projections (99). However, a post-mortem study of ADCY5-related movement disorder did not demonstrate significant dopaminergic neuronal loss, only mild pallor of the substantia nigra (100). Neuropathological post-mortem analyses of Leigh syndrome describe the formation of bilaterally symmetrical lesions predominantly affecting neurons within the basal ganglia and brainstem, particularly regions with high metabolic demand (101).

Finally, neuropathological studies of Rett syndrome (RTT) indicate affected regions include layer III and V pyramidal neurons in frontal, motor, and temporal cortices, which show reduced dendritic arborisation and spine density, alongside alterations in synaptic markers and MAP-2 expression. Reduced nigral neuromelanin content and abnormalities in striatonigral and serotonergic pathways have also been reported, indicating involvement of monoaminergic systems within basal ganglia and brainstem circuits (102).

## PMDs patient iPSC-derived regionally patterned 2D cultures

iPSC-derived 2D neuronal cultures remain widely used within modeling PMDs due to their scalability, reproducibility, and compatibility with high-throughput screening platforms and readouts. Directed differentiation protocols enable the generation of neuronal subtypes selectively implicated in specific PMDs, enabling precise mechanistic or therapeutic studies in a relevant cellular system. Such protocols support the generation of diverse neuronal populations, including excitatory glutamatergic neurons (103–105), inhibitory GABAergic neurons (106, 107), and motor neurons (108–111), as well as supporting glial populations such as astrocytes and oligodendrocytes (112–114). These systems are also amenable to targeted genetic manipulation, including CRISPR-based correction of pathogenic variants for mechanistic validation, as well as optogenetic and other functional genetic engineering approaches to interrogate neuronal activity (115, 116).

2D neurons can be maintained either in isolation or within co-culture systems that incorporate multiple neuronal subtypes and/or include glial cells to enhance physiological relevance. For example, co-culturing iPSC-derived neurons with astrocytes enhances synapse formation, increases neuronal branching, and promotes the establishment of tripartite synaptic structures (117). Similarly, co-culturing neurons with microglia allows the investigation of neuroimmune interactions and the effects of microglia on neuronal health, activity, and network maturation, including non-cell autonomous mechanisms implicated in disorders such as Amyotrophic lateral sclerosis (ALS) and related motor conditions (118).

Microglia, the resident immune cells of the central nervous system (CNS), play essential roles in neurodevelopment, including the regulation of neuronal survival, programmed cell death, synapse formation, and circuit refinement (119–125). Disruption of these microglial-dependent processes is increasingly implicated in neurodevelopmental disorders, where aberrant motor control circuits can arise from early defects in neuronal connectivity and network refinement. Consistent with this, mouse models of Rett syndrome (RTT) show microglia with altered transcriptional profiles, elevated pro-inflammatory and neurotoxic signaling, and a progressive reduction in microglial cell number as disease advances (126–128). Incorporation of microglia and other relevant neural cell types into 2D cultures therefore enables the study of critical developmental processes, including synapse formation, astrocyte-mediated metabolic support, and neuron-glial signaling, all of which have been implicated in the pathophysiology of various PMDs (129–133).

The ease of manipulation, and scalability of iPSC-derived neuronal cultures enable systematic interrogation of patient-specific genetic backgrounds and longitudinal analysis of disease-relevant cellular phenotypes, including intrinsic excitability, synaptic development, mitochondrial function, axonal transport, and proteostasis. Their 2D architecture provides direct physical and optical access to neurons, facilitating high-resolution live-cell imaging, electrophysiological recordings, calcium and voltage imaging, and quantitative image-based assays. These features also make 2D cultures highly compatible with automated screening approaches for small molecules, ASOs, and other therapeutic strategies (134, 135).

Despite these advantages, 2D iPSC-derived neuronal cultures lack the complex 3D cytoarchitecture and ECM interactions that regulate cell polarity, migration, and network formation *in vivo*, limiting their physiological relevance. Although iPSC differentiation broadly recapitulates human neurodevelopment and yields neurons capable of synapse formation and action potential firing, accumulating transcriptomic analyses indicate that these neurons typically remain developmentally immature, resembling fetal-stage rather than adult neuronal profiles (136–139). This immaturity reflects both protocol-dependent constraints on *in vivo* maturation and the resetting of cellular aging signatures during somatic reprogramming (140). Ongoing efforts are actively focused on enhancing neuronal maturation by optimizing differentiation protocols and culture conditions (104, 141, 142). Nevertheless, iPSC-derived neuronal models provide a powerful platform for dissecting the molecular and cellular mechanisms underlying PMDs and for identifying candidate therapeutic interventions.

In disorders affecting dopaminergic neurotransmission, such as AADC deficiency, tyrosine hydroxylase deficiency (THD), and dopamine transporter deficiency syndrome (DTDS), patient-derived midbrain dopaminergic (mDA) neurons have proven particularly informative. For example, AADC patient-derived mDA neurons faithfully recapitulate near-absent AADC enzymatic activity, reduced dopamine metabolites, and severe neurodevelopmental defects, including impaired synaptic maturation, reduced neurite branching, altered neuronal capacitance, and abnormal electrophysiological properties. This model was used to evaluate lentiviral delivery of wild-type *DDC*, restoring AADC activity, normalizing dopamine metabolism, enhancing synaptic protein expression, increasing neurite complexity, and partially reversing neuronal maturation phenotypes. Importantly, modeling of distinct *DDC* variants revealed variant-specific residual enzyme activity and differential responses to levodopa (L-DOPA), establishing a functional framework for stratifying dopamine responsiveness and informing personalized therapy already in clinical practice (143).

Similarly, in THD, iPSC-derived mDA neurons replicate key biochemical and morphological features observed *in vivo*, including reduced intracellular dopamine levels, reduced TH expression, altered dopaminergic marker expression, and pronounced neurite arborisation defects (144, 145). Interestingly, neurons from patients with mild (THDA) and severe (THDB) forms of the disorder demonstrated differential responses to L-Dopa supplementation: treatment restored dopaminergic function and neuronal morphology in THDA neurons, whereas THDB neurons exhibited limited responsiveness unless L-Dopa was supplemented during the neuronal precursor stage (144). This study highlighted a critical developmental window for therapeutic efficacy, a phenomenon that can be uniquely investigated in human models that permit molecular and cellular analysis across neurodevelopmental stages.

Furthermore, 2D neuronal models have been shown to accurately replicate dopaminergic degeneration, a key pathogenic hallmark observed in numerous parkinsonian-like PMDs. For example, DTDS is caused by biallelic loss of function in the dopamine transporter protein, encoded by *SLC6A3*, causing severe progressive childhood Parkinsonism (146). A patient-derived mDA neuron model of DTDS exhibited the expected impairment in dopamine uptake and progressive apoptotic neurodegeneration. Lentiviral delivery of a human *SLC6A3* construct to mDA neurons restored dopamine uptake and rescued neuronal survival, demonstrating *in vitro* proof of concept for gene therapy. These findings were subsequently validated in a knockout mouse model, where stereotactic midbrain delivery of the same construct rescued both survival and motor function (147). The use of a patient-derived neuronal model together with *in vivo* evaluation of motor phenotype rescue established a robust preclinical foundation for subsequent clinical translation.

iPSC-derived projection neuron models have also provided insight into axonopathy-driven PMDs. In spastic paraplegia type 5 (SPG5), biallelic mutations in *CYP7B1* lead to progressive degeneration of corticospinal motor neuron axons leading to lower-limb spasticity (148). Patient-derived cortical projection neurons exhibit impaired axonal outgrowth, axonal swellings, neurofilament disorganization, and disturbances in cholesterol metabolism (149). Given the established clinical use of chenodeoxycholic acid (CDCA) in cerebrotendinous xanthomatosis (CTX), a cholesterol storage disorder, this model was used to assess both the effectiveness of repurposing CDCA for SPG5 and the underlying mechanisms of its neuroprotective effects in both conditions (150). CDCA treatment was shown to rescue axonal degeneration in SPG5 and CTX cortical neurons, providing mechanistic insight into its neuroprotective effects and supporting the translational feasibility of CDCA as a therapeutic strategy for SPG5 (29).

Patient-derived neuronal models are additionally particularly powerful for studying ultra-rare PMDs characterized by early neurodevelopmental disruption, where access to fetal patient tissue is otherwise extremely limited. In GNAO1-related disorders, caused by heterozygous pathogenic variants in *GNAO1*, patients present with a spectrum of neurological phenotypes, including developmental delay, movement disorders, and epileptic encephalopathy (151). Patient-derived dorsal root ganglion (DRG) neurons revealed aberrant proliferation, altered neuronal differentiation, and dysregulated cAMP signaling (152). These findings are consistent with observations from a patient-derived cortical neuronal model, which identified aberrant cell-fate commitment, premature and defective differentiation, altered expression of early neural and astrocytic markers, reduced basal intracellular calcium, and impaired spontaneous neuronal activity (153). DRG neurons were further used to validate a novel allele-specific ASO therapy, designed to selectively reduce expression of the mutated *GNAO1* allele. Treatment with lead ASOs restored cAMP homeostasis and normalized cellular proliferation, providing functional rescue of disease phenotypes in a human genetic context and supporting its potential as a clinically translatable therapy (152).

Finally, iPSC-derived cortical neuron models have proven valuable for investigations into variant-specific disease mechanisms and differential cellular responses to potential therapeutics. In SCN8A-related epilepsy, iPSC-derived excitatory cortical neurons generated from three patients with distinct *SCN8A* variants exhibited variant-specific sodium current abnormalities. Two patients showed elevated, persistent sodium currents, while one showed increased resurgent currents. All patient-derived neurons exhibited shortened axon initial segments (AIS), and prolonged action potential repolarisation leading to hyperexcitability. Network-level analyses revealed that hyperactive burst firing in these neurons was selectively suppressed by the sodium channel–targeting drugs phenytoin and riluzole. Guided by these findings, two of the patients were treated off-label with riluzole, an Food and drug administration (FDA)-approved therapy for ALS, resulting in substantial reductions in seizure frequency (154).

### Directly reprogrammed neurons

While iPSC technology enables scalable production of human neurons, reprogramming through a pluripotent state resets cellular aging hallmarks, including epigenetic age, mitochondrial activity and telomere length (140, 155, 156). As a result, iPSC-derived neurons resemble embryonic or fetal neurons (137–139). This can affect the recapitulation of pathophysiological phenotypes appearing later in disease progression or in late-onset disease. Direct reprogramming offers an alternative approach by converting somatic cells into induced neurons (iNs) without conversion to a pluripotent intermediate state. Consequently, iNs have demonstrated the retention of some age-associated DNA methylation patterns/DNA damage, mitochondrial dysfunction, proteostasis decline, nuclear pore leakage, and disease-relevant phenotypes that may be absent or attenuated in iPSC-derived neurons. However, there is ongoing debate as to whether the full epigenetic state is retained during the reprogramming process (68, 157–160). Though most studies utilizing iNs focus on late-onset neurodegenerative diseases such as Parkinson's and Alzheimer's, this method is particularly relevant for modeling the late-onset or neurodegenerative components of PMDs.

iNs can be generated relatively rapidly through the forced expression of neuron-specific transcription factors, such as BRN2, ASCL1, and MYT1L (the “BAM” factors), which activate pro-neuronal gene programs and rewire the epigenetic landscape to establish a neuronal identity (161). Additional modifications, including small molecules or subtype-specific transcription factors, can further guide iNs toward specific neuronal subtypes relevant for disease modeling, such as dopaminergic or GABAergic neurons (158, 162).

However, compared to iPSC-derived neurons, iNs are limited in scalability and developmental plasticity as they bypass the proliferative progenitor stages, acquiring a post-mitotic state (163–165). This additionally constrains their utility for modeling early neurodevelopmental processes and lineage specification. Furthermore, iNs frequently exhibit variability in conversion efficiency, subtype fidelity, and functional maturation, which limits reproducibility across studies (159, 166).

Despite these limitations, directly reprogrammed neurons provide clear advantages in disease contexts where preservation of age-associated, epigenetic, or mitochondrial features is critical. In myoclonic epilepsy and ragged red fibers (MERRF), a rare mitochondrial primary movement disorder caused by pathogenic mutations in mitochondrial DNA, disease severity is linked to mitochondrial heteroplasmy, with a higher percentage of mutated mtDNA correlating with more severe clinical phenotypes (167). To preserve this feature, direct reprogramming was used to generate iNs that maintain patient-specific heteroplasmy levels, providing a more physiologically relevant model than iPSC-derived neurons in which mtDNA heteroplasmy can be partially reset or selected against (168, 169). MERRF iNs exhibited clear neuronal and mitochondrial pathology, including reduced neurite outgrowth, decreased mitochondrial membrane potential, fragmented mitochondrial networks, elevated reactive oxygen species, and impaired autophagy. This model identified a broad spectrum of disease-related phenotypes and established the efficacy of rapamycin in rescuing mitochondrial dysfunction, an FDA-approved drug that was previously shown to increase mitochondrial biogenesis in patient fibroblasts (170).

iNs also hold substantial advantages when modeling disorders driven by epigenetic dysregulation. Rett Syndrome (RTT) is caused by mutations in the X-linked methyl-CpG-binding protein 2 (*MECP2*) gene, a global regulator of transcription (171). The reprogramming process involved in iPSC-based models resets X-chromosome inactivation (XCI) in female patients, potentially impairing the recapitulation of cellular phenotypes central to pathology (172). Direct reprogramming preserves both XCI patterns and epigenetic signatures, essential for a disease in which MECP2, a methylation-dependent transcriptional modulator, is mutated and dysfunctional. Compared to controls, patient iNs exhibited reduced dendritic arborization and VGLUT1 puncta, downregulated glutamate receptor genes, and hyperacetylation of histones H3K9 and H4K16, reflecting aspects of known RTT neuropathology (173–175). Treatment with a cell-penetrating TAT-MeCP2 fusion protein normalized H4K16 hyperacetylation but did not rescue dendritic morphology over the short term (173). The authors therefore established the therapeutic potential of protein replacement therapy for RTT and a suitable model to further investigate DNA methylation patterns and pathological mechanisms.

The importance of retaining age-associated cellular features is also evident in FA, a progressive multisystem neurodegenerative disorder arising from accumulative mitochondrial dysfunction, iron accumulation, and oxidative stress (176–179). By preserving these progressive pathological features, iNs generated from FA patient fibroblasts offer a potentially more physiologically relevant platform for evaluating potential therapies. Patient-derived iNs were shown to demonstrate reduced frataxin protein levels and significant iron accumulation. Treatment with Alpha-lipoic Acid (ALA) increased frataxin expression levels in patient iN lines and significantly reduced intracellular iron accumulation, establishing the therapeutic potential of ALA for FA (180).

Finally, directly reprogrammed neurons offer particular advantages in disorders with incomplete penetrance for which genetic background has important effects, such as DYT1–TOR1A dystonia. DYT1 dystonia is caused by a heterozygous mutation in the *TOR1A* gene, which encodes the AAA+ ATPase Torsin A. The most common mutation, a GAG deletion in exon 5, only has 30% penetrance and considerable inter-individual variability in clinical presentation (181–183). In such contexts, preserving patient-specific genetic backgrounds, including epigenetic and regulatory states, may be critical for accurately recapitulating disease-associated effects. Reprogramming to pluripotency involves widespread transcriptomic changes with extensive chromatin remodeling, which may attenuate or obscure disease-relevant cellular phenotypes. Consistent with this, a direct comparison of iPSC-derived motor neurons (iMNs) and directly reprogrammed motor neurons (diMNs) allowed the analysis of more pronounced disease-associated defects in diMNs compared to iMNs, including reduced neurite outgrowth, abnormal nuclear morphology, and impaired nucleocytoplasmic mRNA and protein transport. This platform further identified pathogenic overexpression of LMNB1, a nuclear scaffolding protein, in DYT1 neurons and demonstrated that shRNA-mediated LMNB1 knockdown restored nuclear morphology, improved neurite growth and branching, and rescued nuclear export defects (184).

## Three-dimensional organoids to model PMDs

Organoids are self-organizing, multicellular 3D structures derived pluripotent stem cells or tissue-resident stem and progenitor cells. Unlike traditional 2D cultures, organoids allow the recapitulation of essential aspects of human brain cytoarchitecture, including spatial organization, progenitor zones, cortical layering, intercellular interactions, and biomechanical cues, allowing the study of early neurodevelopmental processes and how their alteration may give rise to motor circuit dysfunction.

The generation of organoids can be broadly classified into unguided and guided approaches. Unguided protocols leverage the intrinsic self-organizing capacity of stem cells to form neural tissue when cultured as 3D aggregates, producing heterogeneous, multi-regional structures (185–187). Lancaster et al. expanded upon these findings by embedding human PSCs in Matrigel to mimic the microenvironment of neural development, enabling the self-guided formation of neuroepithelial buds that recapitulate the germinal zones of neural stem and progenitor cells. These cerebral organoids spontaneously formed neural rosettes and cortical-like layers, along with features resembling several brain regions, including the retina, choroid plexus, midbrain-hindbrain boundary, ventral forebrain, hippocampus, and dorsal cortex (188, 189). Unguided cerebral organoids hold great utility in modeling interactions between different brain regions, however, they are limited by substantial batch-to-batch variability, limited reproducibility and the inability to study region-specific processes (190).

In contrast, guided protocols use defined combinations of small molecules and growth factors to pattern pluripotent stem cells toward specific neural lineages, enabling the reproducible formation of cortical (191), striatal (192), midbrain (193), hippocampal (194), spinal cord (195), choroid plexus (196), retinal (197), and many other region-specific structures. Crucially, this regional and cellular specificity allows the interrogation of neuronal subtype-restricted disease mechanisms, including isoform- or cell-type-specific expression of disease-associated genes, differential vulnerability of neural populations, and circuit-level pathology that would be difficult to study in heterogeneous or non-patterned culture systems.

Beyond early development, a major advantage of human brain organoids over 2D cultures is their capacity for extended *in vitro* maturation, enabling neurons to develop over months to years in a manner that more closely parallels postnatal human brain development (198, 199). A recent study from the Arlotta group cultured cortical organoids for an unprecedented 5 years, demonstrating progressive transcriptional and epigenetic maturation that closely tracked predicted epigenetic age and recapitulated sequential *in vivo* developmental programs. Throughout the extended culture, organoids maintained global epigenetic stability, indicating that neurons not only survive long-term *in vitro* but continue to record the passage of developmental time. Importantly, prolonged organoid culture supported the emergence of late-born excitatory neuron subtypes, dendritic spine formation, and functional network activity, highlighting the potential of organoids to model later stages of human cortical maturation (200).

Accordingly, understanding the temporal maturation of brain organoid models is increasingly critical for disease modeling and therapeutic development. Mapping the developmental trajectories of *in vitro* organoids to their *in vivo* counterparts allows disease phenotypes to be interpreted within the appropriate spatial and temporal context of human brain development (191, 201–204). This is particularly important for pediatric disorders, where the timing of intervention can be as crucial as the treatment itself (12). The ability to generate region-specific organoids that reach a defined genetic and epigenetic age, matching the onset or treatment window of a given disorder, would provide a powerful platform for testing therapeutics in a more physiologically relevant context.

Despite these strengths, organoids remain limited by substantial variability in size, cellular composition, and maturation state, which reduces statistical power and reproducibility (205). This intra- and inter-laboratory variability arises from differences in iPSC lines, intrinsic heterogeneity in stem cell morphology and handling, and technical factors within organoid culture workflows, including matrix composition and bioreactor conditions. To advance the robustness of organoid-based research, recent consensus efforts emphasize the need for rigorous quality control of pluripotent stem cell lines, systematic characterization of cellular composition and functional maturity, transparent reporting of protocols and metadata, and benchmarking against human developmental reference atlases (206). Adoption of shared best-practice guidelines, alongside open data and protocol sharing, will be essential to improve reproducibility across laboratories and to establish organoids as reliable, quantitative platforms for disease modeling and drug discovery.

In a series of studies, the Ciani group has exemplified how animal and patient-derived organoid models can be used in a complementary manner to address distinct aspects of disease biology. CDKL5 deficiency disorder (CDD) is a developmental and epileptic encephalopathy (DEE) caused by pathogenic variants in the *CDKL5* gene, which encodes a serine-threonine kinase essential for brain development. *Cdkl5* knockout mouse models were instrumental in establishing the *in vivo* efficacy of a secretable, cell-penetrating TATk-CDKL5 fusion protein, showing partial rescue of behavioral deficits *in vivo* (207). However, because these models do not fully recapitulate seizure phenotypes, patient-derived cortical organoids (COs) were subsequently employed to interrogate human-specific electrophysiological abnormalities. These organoids reproduced key pathological features, including reduced progenitor proliferation, increased neuronal apoptosis, abnormal neuronal morphology, and, critically, network hyperexcitability, providing a quantifiable electrophysiological correlate of seizure-like activity. Viral delivery of TATk-CDKL5 in COs restored proliferation markers, improved neuronal survival, and partially normalized electrophysiological activity, supporting the translational potential of this approach across both murine and human-derived models (208).

Complex patient-derived models also demonstrate significant utility for diseases where mechanistic investigation has been extremely limited by poor access to patient tissue and the absence of suitable animal models. UBA5-associated encephalopathy (DEE44) is an ultra-rare neurodevelopmental disorder caused by pathogenic variants in *UBA5* (DEE44, OMIM: 617132). *Uba5* knockout is embryonically lethal in mice, while viable non-mammalian models fail to recapitulate the hypomorphic genotypes observed in patients (209, 210). Clinically, DEE44 manifests as hypotonia, microcephaly, developmental delay, and epilepsy (211). Patient-derived COs revealed reduced UBA5 expression, impaired UFMylation, endoplasmic reticulum (ER) stress, loss of GABAergic interneurons, and network hyperexcitability. Importantly, this model was used to validate two therapeutic approaches: short interspersed nuclear UP regulation (SINEUP), which are antisense long noncoding RNAs that selectively enhance translation of UBA5 mRNA, and CRISPRa-dCas9, which increases *UBA5* transcription by recruiting activator proteins to its promoter. Both methods modestly raised UBA5 protein levels and UFMylation activity, reduced ER dyshomeostasis, and transiently corrected the abnormal neuronal firing in patient-derived COs (68).

Similarly, animal models of Leigh Syndrome (LS), a severe mitochondrial PMD caused by mutations in *SURF1*, display milder or distinct neurological phenotypes and can fail to reproduce cytochrome c oxidase (COX) deficiencies observed in human patients (212–214). However, patient-derived cerebral organoids showed defects of COX assembly, impaired mitochondrial respiration, disrupted neuronal maturation, and aberrant cytoarchitecture. A combination of single-cell transcriptomics and multi-omics analyses revealed *SURF1* mutations impaired mitochondrial respiration, preventing neural progenitor cells (NPCs) from undergoing the metabolic shift to oxidative phosphorylation (OXPHOS) normally associated with neuronal maturation, thereby locking them in a proliferative, glycolytic state that disrupts proper neuronal morphogenesis. In addition to providing a molecular mechanism by which mutated *SURF1* causes pathology, the authors established two novel treatment avenues for LS by testing on 2D patient-derived NPC models: adeno-associated virus (AAV) delivery of WT-*SURF1*, and pharmacological stimulation of mitochondrial biogenesis through upregulating *PGC1A* transcription with Bezafibrate. Both approaches enhanced OXPHOS metabolism, reduced proliferation, and supported neuronal morphogenesis, establishing two alternative mechanism-based therapeutic strategies for LS (215).

Patient-derived COs, owing to their increased complexity and 3D cytoarchitecture have additionally revealed spatial organization abnormalities in GNAO1-related disorders that were not apparent in 2D cultures. We previously discussed the use of 2D patient-derived neuronal models of GNAO1-related disorders to screen for and evaluate the efficacy of allele-specific ASOs, as well as to identify cellular phenotypes, including aberrant proliferation and altered neuronal differentiation (152). Patient-derived COs, in contrast, revealed severe defects in ventricular zone (VZ) and cortical plate (CP) organization, disrupted neuronal differentiation, and loss of polarized growth cone formation. This system also exposed layer-specific signaling abnormalities, the normal gradient of myosin light chain 2 phosphorylation (pMLC2), which is natively high in the CP and low in the VZ, was abolished in patient COs, indicating hyperactivation of the Rho kinase pathway and dysregulated cytoskeletal signaling. Treatment with the Rho kinase inhibitor Y27632 partially restored VZ circularity, re-established the pMLC2 gradient, and rescued growth cone morphology. This study emphasizes the utility of 3D models to reveal early spatial neurodevelopmental defects (216).

Finally, Tourette's syndrome (TS) provides a useful example of how regionally patterned organoid systems can be used to investigate neurodevelopmental circuit dysregulation arising from complex, polygenic, and epigenetically modulated mechanisms rather than single-gene defects. Although TS is highly heritable, its risk is distributed across numerous common and rare variants in the same individual, each with an additive effect, resulting in substantial genetic and phenotypic heterogeneity between patients (217). Disease manifestation is therefore thought to arise from the interaction of polygenic risk with patient-specific neurodevelopmental trajectories of basal ganglia-cortical circuitry, potentially shaped by epigenetic regulation (218). TS basal ganglia organoids (BGOs) exhibited abnormal ventromedial patterning, with reduced NKX2.1^+^ progenitors, impaired differentiation of GABAergic and cholinergic interneurons, and transcriptional mispatterning of ventral telencephalic progenitors toward dorsolateral and cortical identities. In parallel, TS COs displayed overlapping transcriptional abnormalities indicative of disrupted interneuron development. Mechanistic studies, including early transcriptional profiling, GLI2/3 protein quantification, and primary cilia imaging, showed early dysregulation of cilia-dependent Sonic Hedgehog (SHH) signaling, in which defective primary cilia led to elevated GLI2/3 repressor activity and altered SHH target gene expression. COs and BGOs, therefore, enabled the investigation into a developmental mechanism for TS by which ventral telencephalic identity is blunted and interneuron specification is impaired, highlighting candidate pathways for therapeutic intervention (219).

## Complex network models of PMDs: the advent of assembloid systems

An important advance beyond single organoid systems is the development of assembloids, in which two or more regionally patterned organoids are fused to enable neural interactions and synaptic connections between differential cell populations. Assembloids are able to recreate key features of neurodevelopment, including axonal pathfinding, neuron migration, and aspects of circuit formation/maturation, making them particularly valuable for modeling neurological diseases in which these processes are implicated.

Numerous assembloid models have been generated, each tailored to reconstruct key circuits and recreate neurodevelopmental processes. Dorsal-ventral forebrain assembloids capture the migration of GABAergic interneurons and their functional integration into cortical circuits (220). Thalamocortical assembloids, generated by fusing thalamic and cortical organoids, recapitulate reciprocal glutamatergic projections, enabling studies of sensory-motor processing and synaptic plasticity (221). Striatal-midbrain assembloids recreate basal ganglia circuits, including striatonigral and nigrostriatal loops, enabling the study of systematic dopaminergic modulation (222). These pathways are critical for regulating motivated behavior, voluntary movement, and motor learning, thus this model is often employed for investigating disorders that affect decisive movement (222–224).

To capture increasingly complex neural circuits, researchers have begun fusing more than two regionally patterned organoids. Pioneered by the Paşca group, this includes multi-part assembloids such as a cortico-motor model integrating cortical, spinal, and skeletal muscle organoids to enable in-depth mechanistic studies of motor disorders (225). Building on this, the group established a spinothalamic assembloid, fusing somatosensory, spinal, thalamic, and cortical organoids to recapitulate functional connectivity along the entire sensory-motor axis, thus enabling the recreation of the ascending sensory pathway (395).

Assembloids represent a particularly powerful platform for investigating and translating PMDs, which typically emerge from disruptions across highly interconnected neural circuits rather than from isolated cellular deficits. Motor control depends on the coordinated activity of diverse neuronal populations linked by long-range projections, a level of circuit complexity that is more faithfully captured by assembloid systems than by single-region models. Importantly, functional innervation and subsequent region-specific neurotransmitter release within assembloids enable disease-relevant neuronal function to be studied in response to physiological stimuli or modulatory effects. This ability to recreate coordinated, long-range circuit activity makes assembloids particularly well-suited for modeling disorders in which gene mutations disrupt synaptic connectivity and neuronal communication across interconnected networks. Particularly, cortico-striatal assembloids recreate physiological cortical glutamatergic excitation of the striatum, an essential circuit for motor control, and have been used to investigate the pathophysiology underlying motor phenotypes in diseases such as SCN2A-related disease and *22q13.3* Deletion Syndrome (Phelan-McDermid Syndrome, PS) (192, 226).

*SCN2A* encodes the NaV1.2 channel, with pathogenic variants implicated in a range of neurodevelopmental disorders, including early-onset epilepsy, autism spectrum disorders, and developmental delay (227). A cortico-striatal assembloid model of -related disease demonstrated impaired cortical innervation of the striatum, including reduced dendritic spine density, abnormal spine morphology, and weakened synaptic transmission in recipient striatal medium spiny neurons (MSNs). Molecular analyses revealed downregulation of axon- and synapse-related genes, including voltage-gated sodium channels and synaptic scaffolding proteins, alongside upregulation of nonsense-mediated decay pathways, providing mechanistic insight into *SCN2A* mutation-driven neurodevelopmental pathology (226).

Importantly, this platform identified phenotypes that differ from animal and simpler cellular models. In human assembloids, heterozygous *SCN2A* nonsense mutations (~50% reduction in NaV1.2) result in intrinsic neuronal hyperexcitability, a phenotype that only emerges in mouse models with more severe (>70%) *Scn2a* reduction, highlighting fundamental species-specific differences in neuronal and circuit physiology (226, 228, 229). Moreover, 2D patient-derived cortical neurons display increased AIS length and reduced excitability, though 3D patient-derived assembloids exhibit shortened AIS and network hyperexcitability (226, 230). The 2D cortical model was further used to validate the efficacy of CRISPRa-mediated upregulation of *SCN2A*, which was sufficient to restore both AIS length and electrophysiological activity, directly linking *SCN2A* haploinsufficiency to these phenotypes (230).

The importance of functional innervation and the reconstruction of higher-order neuronal circuitry in revealing physiologically relevant cellular phenotypes is further highlighted by a cortico-striatal model of PS. PS is a neurodevelopmental disorder frequently accompanied by secondary motor phenotypes, including hypotonia, delayed motor development, and abnormal movement patterns, which are thought to arise from dysfunction and degeneration within cortico-striatal circuits (231). Notably, increased calcium spiking was observed in patient-derived striatal organoid MSNs only after their assembly with cortical organoids, indicating a circuit-dependent phenotype. In addition, patient-derived cultures exhibited reduced network synchronization in both striatal organoids and assembloids (192).

In addition to more accurately modeling neural circuits, the other primary advantage of assembloids lies within their capacity to model early neurodevelopmental processes that shape network formation. Forebrain assembloids, generated by fusing independently patterned dorsal and ventral organoids, provide a platform to study the tangential migration of cortical interneurons from the ventral to dorsal forebrain, a process critical for establishing balanced cortical networks (220, 396). In RTT syndrome, this model revealed that *MECP2* mutations drive premature differentiation of neural progenitors in dorsal organoids, depleting intermediate progenitors and outer radial glial cells leading to functional deficits in mature neurons, including immature electrophysiological properties, reduced action potential firing, altered calcium signaling, and decreased synaptic density. In ventral organoids, *MECP2* mutations altered the neurogenesis of medial ganglionic eminence (MGE) progenitors, resulting in fewer migrating interneurons. When fused into assembloids, ventral RTT interneurons exhibited shorter migration distances and inefficient integration into dorsal cortical regions (232).

However, despite holding significant advantages over single-region organoid models, assembloids remain limited by the same challenges surrounding maturity, central necrosis, scalability, and variation.

## Complex multi-lineage assembloid models of PMDs

### Immune-competent neural organoids/assembloids

The functional maturity and physiological relevance of 3D model systems are primarily limited by a lack of vascularisation and the absence of non-neuroectodermal cell types, such as microglia. Given their essential roles in circuit development and maintenance of CNS homeostasis in disease, integrating microglia into *in vitro* models will enhance their ability to more accurately model diseases with neurodevelopmental implications. Consistent with this, the inclusion of microglia in neural organoid cultures has been shown to accelerate neuronal maturation, refine synaptic connectivity, direct axonal outgrowth and cytoskeletal organization, and increase both the synchronization and frequency of action potentials (233–235).

Several strategies have been developed to address this challenge, including co-culture systems, the addition of differentiated microglia, and transcription factor-guided induction. These protocols involve co-culturing organoids with microglial progenitors (234, 236), differentiated microglia (233, 235, 237), or specific molecular manipulations at critical developmental windows to induce spontaneous formation (238, 239). Despite the increasing standardization and reproducibility of neural organoid protocols, the integration of microglia remains technically challenging.

Recent advances have produced fully iPSC-derived immune-competent organoids by integrating microglial and neural precursors, enabling their co-development. Microglia were shown to mature within the organoid system and survive throughout the entire culture period, importantly, without adjustment for microglial-specific media conditions (240). Co-differentiation protocols that generate microglia and neural lineages in parallel have also been shown to yield physiologically relevant microglial proportions (~7%−8%), with microglia exhibiting *in vitro*-like morphology, transcriptomic profiles, and functional behaviors such as phagocytosis, synaptic pruning, and inflammatory responses (241).

Nonetheless, significant limitations remain. The spontaneous formation of microglia within organoid systems is considered to more accurately mimic neurodevelopment, however, these protocols remain less established regarding batch-to-batch heterogeneity and neural-to-glial cell ratios (238, 239). In contrast, introducing partially or fully differentiated microglia at various stages of organoid maturation can result in developmental mismatches that impact microglial maturation, regional identity, and functional integration. These microglia often exhibit immature or non-physiological phenotypes, show uneven infiltration, fail to survive throughout the entire culture period, and may not fully recapitulate *in vivo* behaviors (242–244) Additionally, culture conditions optimized for neurons may be suboptimal for microglia. While immune-competent organoids offer valuable insights into neuron-glia interactions, neuroimmune responses, and developmental circuit formation/maturation, careful validation of microglial maturity and functional integration is necessary.

Microglia-integrated 3D systems are still relatively new within the field, though have already yielded interesting observations regarding the role of microglia in the developing diseased brain. Particularly, a recent study utilized microglia-integrated iPSC-derived cortical (hCO), striatal (hStrO), and midbrain (hMLO) organoids to identify distinctive microglial subtypes: hCOs were enriched for proliferative microglia, hStrOs contained microglia with high GABA_B_ receptor expression and transcriptional programs linked to neuronal excitability, and hMLOs were enriched for immune-responsive microglia (245). The identification of these microglial profiles is particularly significant as while microglia are known to adopt region-specific transcriptional and functional profiles in response to local neuronal identity and circuit activity, this is difficult to investigate in human-relevant systems, particularly in specific disease contexts (246, 247).

This model was further applied to investigate the role of microglia within basal ganglia circuitry development in the context of SCN2A-related disease. Microglia-containing hMLOs and hStrOs were fused to generate striatal-midbrain assembloids, reproducing nigrostriatal circuitry. This model revealed that dopaminergic hMLO neurons trigger robust calcium transients in hStrO microglia upon firing, demonstrating that microglia can dynamically respond to neuronal circuit activity. Furthermore, in patient assembloids, hyperexcitable striatal neurons were shown to induce excessive microglial pruning of inhibitory synapses, as similarly reported in other models (245, 248). The pharmacological inhibition of microglial GABA_B_ receptors reversed aberrant microglial calcium signaling and reduced excessive synaptic engulfment, demonstrating that pathological synapse pruning in this model is mediated by GABA_B_-dependent microglial signaling and providing further insight into disease mechanisms (249).

### Vascularised neural organoids

Lack of a functional vascular network restricts the delivery of oxygen and nutrients to 3D tissues and limits growth, maturation, and long-term viability. 3D *in vitro* models, therefore, depend on passive diffusion, which can only support cells within ~300 μm of the surface and leads to central hypoxia, necrosis, and impaired neural progenitor differentiation in larger models (250). Moreover, the absence of cerebrovascular elements prevents modeling of neurovascular interactions and the development of blood-brain barrier (BBB)-like properties that are essential for assessing therapeutic delivery methods. Vascularisation of 3D *in vitro* models remains a significant challenge due to the different culture requirements of neural and vascular cells, the limited infiltration of vascular networks into dense organoid tissue, and the lack of functional luminal perfusion. Multiple strategies have been developed to address this.

Firstly, genetic engineering strategies have engineered forced expression of the transcription factor ETV2 in iPSCs to drive endothelial lineage specification within cortical organoids, enabling the differentiation of endothelial cells and the formation of robust vascular networks (251). Alternatively, supplementing cerebral organoid cultures with endothelial-promoting factors, such as vascular endothelial growth factor (VEGF), during the embryoid body stage has been shown to drive the formation of vessel-like networks exhibiting mature blood-brain barrier properties (252).

Additional vascularisation strategies involve the introduction of exogenous endothelial cells, either alone or in combination with mesodermal and mural cell precursors, to neural organoids (253, 254). Similarly, the fusion of pre-patterned vascular organoids with brain organoids has emerged as a promising approach to establish more complex, multicellular vascular architectures that better approximate *in vivo* neurovascular organization (255–257). Researchers have also begun combining complex genetic engineering and bioprinting approaches. Neural and endothelial fates can be precisely programmed through inducible expression of lineage-specific transcription factors, enabling their simultaneous differentiation in a single culture system independent of media composition. These genetically engineered iPSCs can then be printed onto complex scaffolds and subsequently differentiated into neural/endothelial cells, enabling the precise control over cell fate, spatial patterning, and tissue architecture (258).

Improvements in the vascularisation of brain organoids not only enhance neuronal function and maturation but also introduce the concept of *in vitro* models of the blood-brain barrier (BBB). The BBB is a highly specialized interface that lines the cerebral vasculature, forming a selective boundary between the circulating blood and neural tissue. It regulates the entry of ions, metabolites, and cells into the central nervous system while limiting exposure to neurotoxic agents and operates as part of the neurovascular unit (NVU) comprising endothelial cells, pericytes, astrocytes, neurons, microglia, and ECM components (259). During early developmental stages, the formation and maturation of the BBB are critical for neurodevelopment, though remain poorly understood in the context of neurodevelopmental disorders and PMDs.

The newborn brain differs markedly from the adult brain, exhibiting a less effective BBB, immature cerebrovascular autoregulation, and higher metabolic demands (41). Elevated glutamate receptor expression and activity during the perinatal period increase the vulnerability of neurons, astrocytes, and oligodendrocytes to excitotoxic injury under conditions of limited energy supply (260, 261). These vulnerabilities underlie the susceptibility of the immature CNS to acute injuries such as hypoxia-ischemia, which can lead to dyskinetic cerebral palsy in infants, though rarely produce comparable motor phenotypes following injury in adulthood (262). Genetic metabolic PMDs, such as GLUT1 deficiency, glutaric aciduria type 1, and LS, underscore the importance of BBB function and vascular-neural interactions during early life, as they primarily present in infancy or childhood. In GLUT1 deficiency, mutations in *SLC2A1* impair glucose transport across the BBB, resulting in cerebral energy deprivation that leads to movement disorders, developmental delay, cognitive impairment, and early-onset epilepsy (263). Thus, there is a need for BBB-integrated organoid models, both to study the mechanisms by which impaired barrier function and neurovascular dysregulation contribute to the etiology of PMDs, and to test therapeutic strategies that rely on effective delivery across the developing BBB.

Current vascularised human brain organoid (vhBO) models provide a promising platform to study early neurovascular interactions and BBB development. vhBOs can form vessel-like structures expressing key tight and adherens junction proteins and are often associated with pericytes, astrocytes, and basement membrane proteins, partially recapitulating features of the NVU (255, 264–266). Key molecular pathways implicated in BBB specification, such as Wnt/β-catenin signaling and retinoic acid metabolism, are enriched in vhBOs compared with non-vascularised organoids, supporting their intrinsic capacity for barrier formation (252, 257). However, endothelial cells within vhBOs remain immature, with limited expression of essential influx and efflux transporters and minimal functional barrier integrity, including permeability and transendothelial electrical resistance (TEER) assessment (254, 267). A major limitation is the lack of physiological perfusion and shear stress, which are critical for endothelial maturation and tight junction organization *in vivo* (268). Though vhBOs capture essential cellular and molecular aspects of early BBB development, current models remain incomplete and immature, emphasizing the need for perfusion-based, microfluidic, and bioengineered systems to achieve functional and mature BBB models for translational applications.

To our knowledge, there are currently no published vascularised human organoid models specifically applied to PMDs. However, the future development of temporally appropriate models of the forming BBB will be critical for studying neurovascular contributions to disease and for evaluating therapeutic delivery strategies, particularly where the BBB may still be developing or functionally affected by disease. Vascularised organoids may be particularly important for modeling PMDs, and other neurological disorders, with progressive pathology, including mitochondrial, metabolic, and neurodegenerative conditions such as Beta-propeller protein-associated neurodegeneration (BPAN), LS, and AT. In such disorders, the ability to sustain organoid maturation over extended periods may be necessary to model later stages of the disease that better correspond to patient pathology, thereby improving the translational relevance of the model system.

### Microfluidics and organ-on-chip

Microfluidics systems are engineered platforms designed to overcome the limitations of nutrient diffusion and replicate the complexity of human neural tissue *in vitro*. Microfluidic designs feature tailored arrangements of microchannels, pores, and chambers to guide cell placement, support growth and migration, and enable the controlled application of biochemical gradients, mechanical forces, and ECM cues to regulate cell behavior (269–273). Brain-on-chip (BoC) technologies leverage microfluidic technologies to model human brain development, function, and disease state under tightly controlled physicochemical conditions, with spatially defined “blood” and “brain” compartments. Early BoC platforms comprised compartmentalized 2D neuronal and glial cultures, enabling directed axonal growth, and the investigation of cell migration (269, 274), synaptogenesis, and immune-neural interactions (275–277) under stable chemical gradients (278). More advanced models integrate complex bioengineering with genetic engineering. For example, Fantuzzi et al. developed a 96-well BoC system with fluidically isolated compartments connected by microchannels, allowing spatial segregation of iPSC-derived excitatory and inhibitory neurons, directed axonal outgrowth, and the formation of intercompartmental synapses. This model enabled the interrogation of excitation-inhibition circuit assembly and synaptic signaling via calcium imaging, chemogenetic-mediated manipulation of neuronal activity, and high-throughput assays to assess synaptogenesis (279).

Integrating microfluidic technology into 3D models enables precise spatiotemporal regulation of the microenvironment, including oxygen tension, mechanical forces, and biochemical gradients (280–282). Compared with organoids cultured alone, BoC systems improve oxygen and nutrient delivery, reduce shear stress, and enhance waste removal, thereby supporting growth and structural maturation (283). A major advance has been achieving functional perfusion of organoids by coupling extrinsic microfluidic flow to a self-organized vascular network within the organoid, though this has not yet been applied to neural systems. Endothelial cells embedded in a 3D hydrogel form microvessels that anastomose with the organoid's intrinsic vasculature, creating a continuous, perfusable network through which oxygen, nutrients, and soluble factors are able to diffuse into the adjacent tissue (284). With regard to neural models, research has demonstrated that embedding COs on a pre-formed vascular network results in mostly peripheral vascularisation (285), though co-differentiation protocols produce full vascularisation of cerebral tissue with more physiologically relevant neurovascular architecture (286).

Faithful *in vitro* modeling of the BBB is critical for PMD translation, not only for studying neurodevelopmental processes and disease mechanisms that disrupt BBB maturation, but for accurately assessing the ability of candidate therapeutics to cross the BBB and reach neural targets. Microfluidic BBB models are the most physiologically relevant *in vitro* systems to date, replicating cellular, mechanical, and biochemical environments. Based on architectural design, microfluidic BBB platforms can be categorized into sandwich, parallel, and 3D tubular configurations. Sandwich models employ porous membranes to separate vascular and neural compartments, allowing co-culture and quantitative permeability measurements, but limiting direct cell-cell interactions (287, 288). Parallel designs replace membranes with micropillar arrays, enabling intercellular communication while also retaining compartmentalisation (289, 290). 3D tubular models recreate capillary-like geometries within hydrogels, producing uniform shear stress and *in vivo*-like endothelial morphology (291–294).

A significant recent advance is the development of a fully patient-derived 3D microfluidic BBB model encompassing iPSC-derived endothelial cells, mural cells, and astrocytes that self-organize into perfusable, vessel-like structures. This model recapitulates key morphological, molecular, and functional features of the human BBB, including tight junction formation, polarized transport, low permeability, and response to inflammatory stimuli (295). Such models could have critical applications in PMDs in which BBB dysfunction, altered transport capacity, or inflammation-driven permeability contribute to disease pathogenesis, including argininosuccinate lyase deficiency (296), GLUT1 deficiency syndrome (297), Sydenham's chorea (298), and PANDAS-associated movement disorders (299).

For example, in RS, where the causative gene *MECP2* is expressed in both neural cells and BBB components such as endothelial cells and pericytes, microfluidic BBB models enable direct investigation of the impact of *MECP2* mutations on neurovascular function that is difficult to capture in conventional cultures. Patients have been shown to exhibit reduced skeletal growth and hypoperfusion in the midbrain and brainstem, reflecting neurodevelopmental vascular abnormalities (300, 301). A patient-derived 3D microfluidic model of RTT vasculature revealed increased permeability, decreased tight junction protein expression, and altered secretion of vascular remodeling factors, indicating compromised barrier function. Transcriptomic and microRNA analyses further identified pathogenic upregulation of endothelial-specific miR-126–3p. Lentiviral delivery of antisense miR-126–3p partially restored endothelial barrier integrity, demonstrating the mechanistic role of miR-126–3p in RTT vascular dysfunction and identifying a novel therapeutic approach (302).

Similarly, microfluidic platforms provide distinct advantages for modeling axon-specific pathology due to the customisable compartmentalisation of cell types, as exemplified in FA. Axonal dystrophy is a core pathological feature of FA that is difficult to interrogate in conventional culture systems but is well-suited to compartmentalized microfluidic models. Patient-derived dorsal root ganglion organoids (DRGOs) were cultured in chambers designed to direct axonal projections through adjacent microgrooves, with a neurotrophic gradient applied to enhance outgrowth. FA axons were found to contain fewer mitochondria of a smaller size, in addition to their increased circularity, indicative of fragmentation. The complexity of this model was advanced further by seeding the DRGOs on intrafusal muscle fibers to investigate whether observed mitochondrial defects affected the ability to form muscle spindles. Patient axons were observed to form significantly fewer muscle spindle-like structures compared to controls. The authors further demonstrated that CRISPR/Cas9-mediated excision of the pathological Guanine-adenosine-adenosine (GAA) expansion and intronic flanking regions of the *FXN* gene restored mitochondrial morphology and density within axons, in addition to correcting deficits in axon-target connectivity (303). By physically isolating axons from neuronal somata, this model enabled axon-specific analysis of disease mechanisms and therapeutic rescue that would be difficult to resolve in non-compartmentalized cultures.

## Discussion

Historically, understanding disease mechanisms and therapeutic development for PMDs relied on animal models and reductionist cellular systems that captured fragments of disease pathology, but failed to fully reproduce human-specific, developmental, and circuit-level mechanisms. The past decade has marked a fundamental shift in how PMDs are modeled, understood, and increasingly treated. This scientific transition has been accelerated by regulatory change. In the United States, the *FDA Modernization Act 2.0* (2022) replaced the requirement for “animal testing” with “nonclinical testing,” explicitly permitting data from human-relevant *in vitro* systems to support investigational new drug applications. Similarly, the UK MHRA has confirmed that animal studies are not legally mandatory when suitably validated data from alternative models are available. These shifts acknowledge concerns surrounding the limited value of animal models to predict human efficacy and toxicity and, importantly, support the establishment of regulatory pathways that allow patient-derived systems to be used as translational evidence (304).

One of the most important conceptual advances enabled by patient-derived platforms is the ability to move beyond a binary “mutation equals disease” framework. Monogenic PMD pathophysiology is shaped by an abundance of factors, including reduced penetrance, genetic modifiers, epigenetic regulation, and developmental timing (23–25). Patient-derived cells retain the full endogenous genetic background of the donor and are therefore critical for understanding mechanisms underlying both disease emergence and resilience. Comparisons between iPSC-derived neurons from affected and non-manifesting carriers have already revealed protective pathways and compensatory mechanisms that are indiscernible in genetically engineered animals or cell lines (39). The continual use of patient-derived models of disease will undoubtedly shed more light on genetic contributors to disease and our understanding of reduced penetrance.

These models have also shown that many PMDs exhibit early disruptions in neurodevelopment. AADC deficiency, TH deficiency, GNAO1, SCN2A, RTT syndrome, and CDKL5 all demonstrate that early defects in neuronal specification, synapse formation, axon targeting, or network maturation may precede and drive later motor phenotypes (66, 143, 152, 153, 173, 207, 208, 230, 232, 249, 305). Human *in vitro* systems uniquely capture this temporal unfolding of disease, revealing critical developmental windows during which treatment is likely to be most effective. The delivery of small molecules, ASOs, or protein replacement therapy can be optimized not just based on genotype, but informed by the experimental determination of optimal timings of intervention. For instance, L-Dopa responsiveness within TH deficiency has been shown to vary across distinct variants and stages of intervention in patient-derived models, thus could potentially inform therapeutic windows when applied clinically (144).

Crucially, patient-derived *in vitro* models are no longer merely descriptive models for improving our mechanistic understanding of disease, they are becoming decision-making tools for therapeutic development. Multiple PMDs now have documented cases in which *in vitro* phenotypic rescue predicted clinical response, including Riluzole for SCN8A epilepsy (306), L-Dopa supplementation in AADC deficiency (143) and CDCA supplementation for SPG5 (307). This represents a paradigm shift from population-based drug development to experimentally guided, patient-specific therapeutic strategy.

The field is also moving beyond purely neuronal models. The incorporation of microglia, vasculature, and BBB components into novel platforms reflects a growing recognition of the need for complex models to model equally complex disorders. Future BoC platforms are expected to evolve into highly integrated systems that combine multiple layers of biological and technological complexity. These next-generation devices will integrate (1) regionally patterned 3D models, (2) microfluidic architectures that deliver physiologically relevant flow and mechanical cues, (3) embedded biosensors that enable real-time monitoring of electrical, metabolic, and molecular activity and (4) engineered biological-synthetic interfaces that reproduce vascular and immune system interactions (308). A further major advance will be the emergence of multiorganoid-on-chip, or “body-on-chip,” platforms in which multiple organ-like systems are connected through microfluidic circulation, enabling the study of interorgan crosstalk and more accurate prediction of drug efficacy and toxicity (309, 310).

Despite the transformative potential of patient-derived *in vitro* systems, several technical and conceptual challenges remain. Patient-derived models are constrained by variability between iPSC lines, differentiation protocols, and laboratory workflows, which can hinder reproducibility and limit systematic comparisons across studies. Standardized benchmarking using single-cell transcriptomics, epigenomics, functional electrophysiology, and the use of isogenic controls will be essential if these systems are to support regulatory decision-making.

Another significant limitation is developmental immaturity. Although this characteristic can facilitate the modeling and understanding of pathological mechanisms during the neurodevelopmental stage of PMDs, it restricts the investigation of later-emerging features, including neurodegeneration, late-onset electrophysiological properties, long-term synaptic plasticity, mitochondrial dysfunction, and metabolic stress. It also presents challenges when assessing therapeutic efficacy, as the system is inherently immature and not representative of *in vivo* neuronal maturity. Approaches such as extended culture, patterned electrical stimulation, metabolic conditioning, vascularisation, and transplantation-based maturation are currently being developed to address this challenge (200, 257, 311–313). In addition, although organoids and assembloids recapitulate key aspects of tissue architecture and regional connectivity, they remain incomplete representations of the human nervous system. These models lack full cellular diversity, myelination, sensory and motor feedback, and fully established long-range circuit integration. Consequently, higher-order features of motor system function are only partially captured.

Notably, transplantation studies have demonstrated that organoids undergo vascularisation, progressive maturation, and functional integration into host neural circuits when introduced during early neurodevelopment (311). This approach has been successfully applied in disease modeling and therapeutic development. For example, transplanted patient-derived organoids have been used to demonstrate that differences in microglial phenotypes in autism are due to a pathological brain environment, rather than inherent microglial genetic predisposition (314). Similarly, COs derived from patients with Timothy Syndrome have been transplanted into the rat brain to model synaptic and dendritic abnormalities, and further used as a platform to evaluate the efficacy of an ASO therapy (315). However, transplantation studies have inherent constraints, including species-specific differences in circuit formation and maturation, differential niches and signaling gradients during neurodevelopment, and ongoing ethical concerns (311, 313, 316).

There are also important translational considerations when moving from patient-specific *in vitro* models toward wider clinical interpretation. The same genetic specificity that makes patient-derived models powerful can also limit their broader applicability. Given the genetic and clinical heterogeneity of PMDs, robust interpretation of *in vitro* phenotypes will require large, clinically annotated iPSC biobanks that support systematic cross-patient comparison and genotype-phenotype stratification. Such resources will be essential for separating disease-relevant phenotypes from background genetic and phenotypic variation and for identifying patterns that recur across patients. In addition to this, *in vitro* assessment of therapeutic efficacy will require numerous patient lines with matched isogenic controls and large “*n*” numbers prior to translational progression. As demonstrated in other rare-disease research frameworks, the long-term translational value of PMD models will depend on the development of shared databases that enable phenotypes to be interpreted across patients and mutations (317, 318).

Where patient-derived iPSCs may not be available, a viable alternative to patient-derived systems is the use of isogenic models derived from standardized healthy control iPSCs, in which pathogenic variants are introduced by CRISPR/Cas0-based gene editing to enable precise attribution of phenotypes to specific genetic lesions (319). This approach is particularly well-suited for mechanistic studies on monogenic disorders, offering strong control over potential genetic background factors, though at the expense of capturing genetic modifiers and variable expressivity.

Importantly, the increasing adoption of patient-derived *in vitro* models does not diminish the continued value of animal models in PMD research, but rather necessitates closer alignment and integration between these platforms to maximize translational impact. While human *in vitro* systems capture human genetic, developmental, and cellular mechanisms, animal models remain essential for interrogating whole-organism physiology, long-range circuit integration, motor behavior, and pharmacokinetics that cannot be fully modeled *in vitro*.

While we have discussed the differences between some animal models and their clinical counterparts, it is important to highlight the animal models that do faithfully recapitulate patient phenotypes. For example, the spinal-restricted *Tor1a* conditional knockout mouse model closely recapitulates severe childhood-onset DYT1-TOR1A dystonia, including early postnatal onset, caudo-rostral progression of symptoms, and fixation of abnormal postures below the head, closely mirroring the human clinical trajectory. Mice also exhibit the physiological hallmarks seen in patients: spontaneous muscle activity at rest, antagonist co-contraction during movement, and impaired monosynaptic reflexes, making this one of the most clinically faithful mouse models of a PMD (31).

Similarly, RTT represents one of the most robust examples of animal-clinical concordance among PMDs. RTT typically begins with a subtle phase of early developmental slowing, in which mild delays in motor and speech development may go unnoticed, often delaying diagnosis. This is followed by a period of rapid regression marked by loss of acquired hand skills and language, worsening motor function, breathing abnormalities, social withdrawal, and the emergence of seizures. Patients then enter a relatively stable phase in which motor impairment and epilepsy persist, while some communication abilities may partially recover. In later stages, progressive motor decline leads to severe physical disability. Canonical *Mecp2*-null and *Mecp2*-mutant mouse models closely mirror this trajectory, showing normal early development followed by neurological regression, impaired mobility, ataxic gait, hindlimb clasping, seizures, reduced brain volume, and shortened lifespan. Importantly, female heterozygous mice also display delayed and variable symptom onset, reflecting X-chromosome inactivation-driven mosaicism observed clinically (320).

Cross-validation between *in vitro* and animal models therefore represents a critical step in translational research. This includes using *in vitro* platforms to identify and prioritize therapeutic interventions with a rescue of cellular phenotypes, followed by targeted *in vivo* testing to assess safety profiles, toxicity, and rescue of behavioral/motor phenotypes.

### Future perspectives

The field is converging toward three priorities. First, increasing biological fidelity through immune-competent and vascularised models will allow PMDs to be studied in environments that more closely mirror the developing human brain. Second, standardization and benchmarking will be essential to make these systems quantitatively reliable for drug development and regulatory use. Third, the continued integration with genomics, single-cell and spatial multi-omics, computational modeling, and scalable CRISPR-based perturbation approaches will shift patient-derived systems from largely descriptive platforms toward mechanistically informed and predictive tools for precision medicine. In particular, CRISPR-enabled functional genomics enables the systematic evaluation of variants of uncertain significance, the identification of genetic modifiers influencing penetrance and disease severity, and the prioritization of therapeutic targets (37, 321, 322).

Patient-derived *in vitro* models are poised to play an increasingly central role in precision pediatric neurology. As regulatory agencies move toward accepting human-relevant non-animal data, these systems are likely to become not only discovery tools but also components of regulatory-grade evidence packages for drug approval and clinical trials.

Current N-of-1 therapies are relatively limited within the literature and utilize patient fibroblasts for the screening and validation of personalized therapeutics. In a landmark study, Milasen, a personalized ASO for CLN7 Batten disease, was screened in patient fibroblasts, tested for safety in rats, and administered within 1 year of starting this process (323). Atipeksen is another example in which a personalized ASO for AT was advanced to human clinical trials after correction of aberrant splicing and functional rescue were demonstrated in patient cells (324). Both therapies demonstrated measurable clinical benefit and further illustrate how patient-specific *in vitro* validation can justify first-in-human dosing and guide therapeutic design in conditions where conventional trials are not feasible. The employment of increasingly complex patient-derived models will transform this field, providing more physiologically relevant systems on which to establish novel personalized therapies and support clinical trials. The recent launch of initiatives such as the Rare Therapies Launchpad further formalizes this paradigm, positioning patient-derived systems as central infrastructure for the development of individualized treatments in rare disease (325). In parallel, the establishment of scalable frameworks for patient-derived organoid production and ASO screening is lowering barriers to personalized intervention, expanding access to precision therapeutics for ultra-rare disorders (87).

Furthermore, for PMDs with a neurodegenerative component, cell-replacement therapy could benefit through engraftment or trophic effects. A number of centers worldwide are developing clinical translatable strategies for cell-based treatment of adult PD (326). Proof-of-principle studies using human fetal ventral mesencephalic allografts showed long-term promising results in PD patients, with an ongoing European Union-funded clinical trial (TRANSEURO, NCT01898390). To date, compassionate treatment using patient iPSC-derived mDA progenitors in an individual with idiopathic PD was conducted in the US (327) and clinical trials with iPSC-derived mDA progenitor cells and embryonic stem cells have started in Sweden and the UK (UMIN000033564, NCT04802733, and STEM-PD, respectively). Clinical trials have already demonstrated success, with BlueRock Therapies' exPDite-2 advancement to a phase III trial after illustrating positive safety and motor outcomes (NCT06944522). Moreover, *in vitro* patient-derived complex systems can be employed to investigate the potential of allogenic and autologous cell-replacement therapy. For example, a 3D assembloid model of the dopaminergic system, comprising midbrain, striatal, and cortical components, was employed to demonstrate the integration of grafted ventral midbrain progenitor cells into the model's existing basal ganglia-like circuitry (328).

While we have discussed gene-based therapies as a promising avenue for PMDs, it is also important to acknowledge significant translational and safety hurdles that currently constrain their success. Gene-replacement therapies are commonly delivered using viral vectors (particularly AAVs), which despite their efficiency, can trigger immune responses and are associated with dose-dependent toxicities, most notably acute liver injury (329, 330). Furthermore, there are concerns surrounding off-target genomic alterations, mosaic editing efficiency, and long-term genotoxic risk (330). These risks were underscored by recent clinical setbacks in Duchenne muscular dystrophy, where multiple cases of fatal acute liver failure were reported following treatment, leading to FDA safety investigations and the suspension or modification of several clinical trials (331). The long-term success of gene-based therapies will depend on advances in vector engineering, delivery precision, safety profiling, and alternative non-viral or RNA-based therapeutic strategies such as ASOs or siRNAs. Importantly, novel delivery approaches, such as stereotactic delivery, are increasing the translational relevance of RNA-based therapeutics that require repeated administration (332).

Finally, and perhaps most importantly, patient-derived platforms are redefining how rare PMDs are conceptualized and treated. Rather than viewing each disorder as the functional consequence of mutations within a singular gene, patient-derived systems reveal how neurodevelopmental circuits adapt to genetic perturbations over time. This perspective supports a shift from static, genotype-based treatment to dynamic, developmentally informed intervention strategies, in which therapies are timed and tailored to the evolving biology of the developing nervous system. In this way, patient-derived *in vitro* models are poised to complement animal disease models, enabling an entirely new framework for understanding and treating PMDs, where genomic and molecular characterization can be directly connected to disease mechanisms and therapeutic response, supporting truly personalized care.

**Figure 1:**
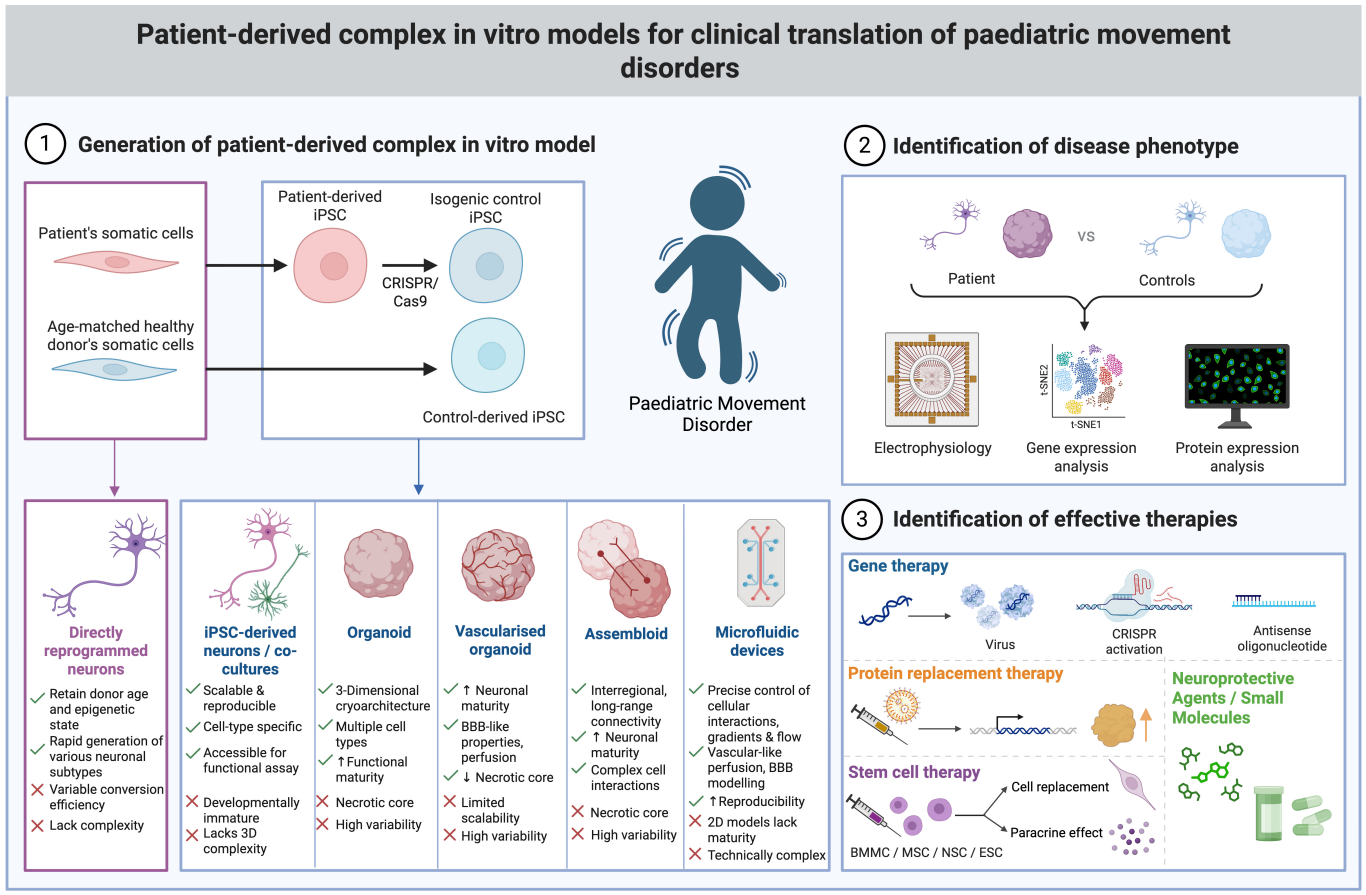
Summary of current state of the art applications of patient-derived *in vitro* models of PMDs. BioRender. Yeadon, S. (2026) https://BioRender.com/bt1bzpx.

To summarize, *in vitro* modeling of PMDs is moving from a proof-of-concept phase to a translational platform for precision pediatric neurology. As these systems continue to advance, patient-derived *in vitro* models are well-positioned to become the central bridge between molecular diagnosis and personalized therapy for children with movement disorders (Figure 1).
